# Damage-dependent regulation of MUS81-EME1 by Fanconi anemia complementation group A protein

**DOI:** 10.1093/nar/gkt975

**Published:** 2013-10-28

**Authors:** Anaid Benitez, Fenghua Yuan, Satoshi Nakajima, Leizhen Wei, Liangyue Qian, Richard Myers, Jennifer J. Hu, Li Lan, Yanbin Zhang

**Affiliations:** ^1^Department of Biochemistry & Molecular Biology, University of Miami Miller School of Medicine, Miami, FL 33136, USA, ^2^Department of Microbiology & Molecular Genetics, University of Pittsburgh, Pittsburgh, PA 15213, USA and ^3^Department of Epidemiology & Public Health, University of Miami Miller School of Medicine, Miami, FL 33136, USA

## Abstract

MUS81-EME1 is a DNA endonuclease involved in replication-coupled repair of DNA interstrand cross-links (ICLs). A prevalent hypothetical role of MUS81-EME1 in ICL repair is to unhook the damage by incising the leading strand at the 3′ side of an ICL lesion. In this study, we report that purified MUS81-EME1 incises DNA at the 5′ side of a psoralen ICL residing in fork structures. Intriguingly, ICL repair protein, Fanconi anemia complementation group A protein (FANCA), greatly enhances MUS81-EME1-mediated ICL incision. On the contrary, FANCA exhibits a two-phase incision regulation when DNA is undamaged or the damage affects only one DNA strand. Studies using truncated FANCA proteins indicate that both the N- and C-moieties of the protein are required for the incision regulation. Using laser-induced psoralen ICL formation in cells, we find that FANCA interacts with and recruits MUS81 to ICL lesions. This report clarifies the incision specificity of MUS81-EME1 on ICL damage and establishes that FANCA regulates the incision activity of MUS81-EME1 in a damage-dependent manner.

## INTRODUCTION

Interstrand cross-links (ICLs) covalently tether both strands of a DNA helix and block essential DNA transactions including replication and transcription. DNA replication is one of the critical factors to elicit repair of ICLs (1–4). Generally, when an ICL blocks the replication machinery, a protein complex termed as the Fanconi anemia core complex is recruited to stalled replication forks and monoubiquitinates another two Fanconi anemia proteins FANCD2 and FANCI. This initiates a string of ICL repair events including damage incision, translesion synthesis and re-establishment of replication forks through homologous recombination (5–10).

Fanconi anemia is a severe genetic disorder characterized by bone marrow failure, developmental defects, chromosomal instability and predisposition to cancer. Fanconi anemia cells are hypersensitive to DNA cross-linking compounds including mitomycin C, cisplatin and diepoxybutane, indicating that they are defective in repairing ICLs (5,11–19). Thus far, 15 distinct genes have been identified to cause the severe disease (17). Although deficiency of each gene shows similar clinical and cellular phenotypes, ∼60% of Fanconi anemia patients presented defective FANCA (9,15), indicating that this protein may have additional biological functions beyond the canonical pathway through FANCI-FANCD2 monoubiquitination. 

Individual components of the Fanconi anemia core complex directly participate in ICL repair as well as in maintenance of replication forks (20–25). FANCA is considered a component of the Fanconi anemia core complex (including FANCA, B, C, E, F, G, L, M and other Fanconi anemia associated proteins (FAAP)). FANCA has been shown to have intrinsic affinity to nucleic acids and has been found to be localized to chromatin in a replication-dependent manner (26–28). FANCA deficient cells clearly showed lower incision of psoralen ICLs compared with wild-type cells and FANCB cells, indicating a specialized role for FANCA in ICL incision (29). Additionally, using nuclear protein extracts and complementation analysis, it was demonstrated that FANCA is required for efficient incisions at the sites of psoralen-mediated ICLs (30). These data imply that FANCA may function outside the Fanconi anemia core complex and directly participate in ICL incision. 

It is well established that ICLs are incised in a replication-dependent manner (1,2,31,32). Several prevalent models propose that two members of the Xeroderma pigmentosum group F protein (XPF) family of DNA endonucleases, XPF-ERCC1 and MUS81-EME1, participate in replication-dependent ICL incision by cutting DNA at the 5′ and 3′ sides of an ICL, respectively (33–36). MUS81-EME1 cleaves 3′ single-stranded DNA (ssDNA) branch and replication fork efficiently (37–47), making it a suitable candidate for ICL incision in replication forks. MUS81-EME1 promotes conversion of ICLs into double strand breaks (DSBs) in a replication-dependent manner (48). Intriguingly, Kanaar and colleagues also found that MUS81 is not involved in the generation of DSBs from DNA damage that affects only one strand of the DNA duplex (48). Collectively, these results indicate that the structure-specific DNA endonuclease MUS81-EME1 is specifically involved in incision of ICLs, but not non-ICL DNA damage, residing in a replication fork. However, it remains unknown how MUS81-EME1 exactly incises the ICL-damaged replication forks and how the incision is regulated to promote ICL unhooking, and how it avoids non-specific incision of undamaged or non–ICL-damaged forks.

In this study, we investigated the ICL incision activity of MUS81-EME1 using purified proteins and a defined site-specific psoralen ICL substrate. We report that MUS81-EME1 incises leading strand at the 5′ side of the ICL. More importantly, we found that FANCA regulates the endonuclease activity of MUS81-EME1 in a damage-dependent manner. 

## MATERIALS AND METHODS

### Expression and purification of human MUS81-EME1 and FANCA

Complementary DNAs for human MUS81, EME1 and FANCA were obtained by polymerase chain reaction amplification from a universal complementary DNA pool (BioChain Institute, Inc.). The full-length open reading frames were confirmed by sequencing and found to exactly match NCBI Reference Sequence NM_025128, NM_152463 and NM_000135, respectively. Co-expression of the hexahistidine-tagged EME1 and non-tagged MUS81 and overexpression of non-tagged FANCA were achieved in insect High Five cells using the Bac-to-Bac expression system (Invitrogen, Carlsbad, CA). Expression of MUS81-EME1, FANCA and its mutants was confirmed by western blot analysis using a Pierce ECL kit (Pierce, Rockford, CA). Antibody against MUS81 was purchased from Novus Biologicals (Littleton, CO). A monoclonal antibody against hexahistidine tag (GenScript, Piscataway, NJ) was also used to confirm EME1 expression and subsequent purification. Antibody against FANCA was kindly provided by the Fanconi Anemia Research Fund. 

Upon expression of MUS81 and His_6_-EME1 in insect cells, the cells were homogenized in a protein extraction buffer (20 mM Hepes–KOH, pH 7.5, 0.5 mM MgCl_2_, 50 mM NaCl, 0.2 M sucrose, 5 mM β-mercaptoethanol, protease inhibitors (0.5 mM PMSF, 0.3 mg/ml benzamidine hydrochloride, 0.5 μg/ml of pepstatin A, 0.5 μg/ml of leupeptin, 0.5 μg/ml of antipain)) by a Dounce homogenizer and 10 strokes on ice. MUS81-EME1 were purified by using a HiTrap chelating column charged with nickel, a Mono S and/or a Superdex 200 gel filtration column (GE Healthcare, Piscataway, NJ) and by tracing MUS81-EME1 protein through SDS–PAGE and western blot. Wild-type (WT)-FANCA and its mutants were purified using a protocol described previously (28). Protein concentration was determined by the Coomassie (Bradford) Protein Assay Reagent (Pierce, Rockford, CA). The purified proteins were stored in −80°C in aliquots.

### Creation of psoralen ICLs

To create a site-specific DNA ICL, a short oligo, 5′-GCTCGGTACCCGG, with an internal psoralen modified T (underlined) was synthesized by Midland Certified Reagent Company (Midland, TX). After elongating through ligation, annealing with a partially complementing oligo, exposing to ultraviolet A irradiation and purifying by denaturing gel (Supplementary Figure S2), we obtained an ICL-damaged splayed arm structure. The splayed arm structure can be labeled at the 3′ end on the top leading strand through α^32^P incorporation by Klenow DNA polymerase (Supplementary Figure S3). Labeling of the 5′ ends of the leading and lagging strands is done before creation of the ICL (Supplementary Figure S3). Through annealing with different oligos complementing to the leading or lagging strand or both, 5′ ssDNA branch, 3′ ssDNA branch and static replication fork structures were created (Supplementary Figure S2).

### DNA incision assay

A total of 1.5 nM of labeled DNA substrate was incubated with indicated amount of proteins in a 10-µl reaction mixture with 25 mM Hepes–KOH, pH 7.6, 1 mM DTT, 3 mM MgCl_2_, 6.5% glycerol, 120 µg/ml BSA and 100 mM KCl. After incubation for 20 min at 37°C, the reaction was terminated by adding 5 µl proteinase K (25 mM EDTA, 0.67% SDS, 150 µg/µl of proteinase K) and 10 min incubation at 37°C, and by adding 15 µl sequencing dye. The reaction mixture was resolved by running a 10% denaturing sequencing gel. DNA size markers were prepared by labeling a mixture of defined oligos through γ-^32^P-adenosine triphosphate.

### RNA interference, induction of ICL in living cells and confocal microscopy

To study the interaction of FANCA and MUS81 in human cells, we treated U2OS cells with ON-TARGET Plus SMART Pool siFANCA (Thermo Scientific Dhamacon, cat# L-019283-00) and a non-targeting control small interfering RNA (siRNA) (cat# D-001810-01-05) using the Dhamafect transfection agent for 48 h. After western blot verification of knockdown efficiency (Figure 6A), a GFP-tagged MUS81 construct, pEGFP-N1-MUS81 (vectors are purchased from Clontech), was transfected into the U2OS cells by Lipofectamine 24 h before drug treatment. pEGFP-N1-FANCA was transfected into WT U2OS cells for monitoring status of FANCA. The Olympus FV1000 confocal microscopy system was used (Cat. F10PRDMYR-1, Olympus, UPCI facility) and FV1000 software was used for acquisition of images. To create psoralen ICLs, cells were pretreated with 100 nM of 8-methoxypsoralen (8-MOP) for 10 min right before a 405 nm laser micro-irradiation. The output power of the laser (original 50 mW) passed through the lens is 5 mW/scan. Laser light passed through a PLAPON 60× oil lens (super chromatic abe. corr. obj W/1.4NA FV, Cat. FM1-U2B990). Cells were incubated at 37°C on a thermo-plate (MATS-U52RA26 for IX81/71/51/70/50; metal insert, HQ control, Cat. OTH-I0126) in Opti-MEM during observation to avoid pH changes. The images were taken 2 min after laser treatment.

## RESULTS

### MUS81-EME1 does not incise on the 3′ side of a psoralen ICL

Although it was proposed that MUS81-EME1 unhooks DNA ICLs by incising the leading strand at the 3′ side of the damage (33–35), it had never been tested how MUS81-EME1 acts on ICL-damaged fork structures. To evaluate how MUS81 incises ICL-damaged DNA, we co-overexpressed human MUS81 and hexahistidine-tagged EME1 in High Five insect cells and purified the MUS81-EME1 complex to near homogeneity (Supplementary Figure S1). Using defined sequence, we also prepared a splayed arm structure with a psoralen ICL located immediately at the junction site of ssDNA and double-stranded DNA (Supplementary Figure S2). The rationale for this design was based on recent observations from Walter’s group (3,4), who reported that ICL incision happens after the ICL damage is within 1 nt of the nascent strand end.

**Figure 1.**
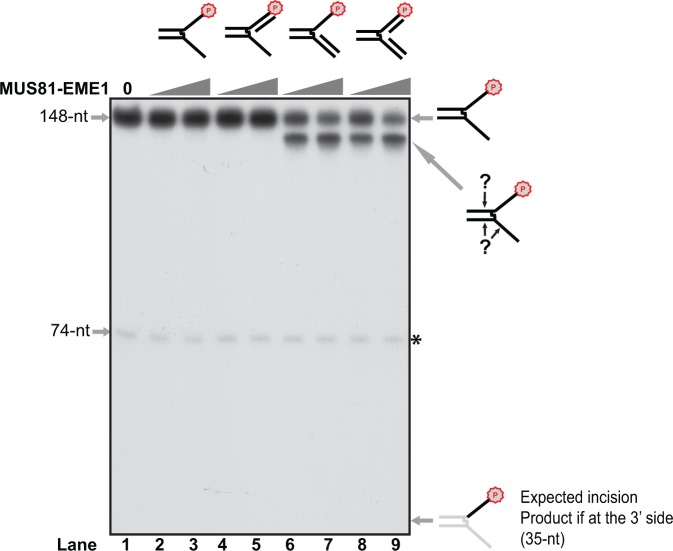
MUS81-EME1 incision on the 3′-labeled ICL-containing DNA structures. Titration of purified MUS81-EME1 (5–10 nM) on the 3′-labeled psoralen ICL-damaged DNA structures as shown on the top. The schematic appearance of the products after incision were shown on the right. All reactions were performed in 10 µl of 25 mM Hepes, pH 7.6, 100 mM KCl, 3 mM MgCl_2_, 1 mM DTT, 120 µg/ml BSA, 6.5% Glycerol and 1 nM of each DNA substrate as indicated. The reactions were performed at 37°C for 20 min and resolved on a 10% denaturing PAGE gel. Letter P with a circle indicates α-^32^P labeling by Klenow DNA polymerase. Asterisk (74 nt) indicates a decayed and uncross-linked species.

To test whether MUS81-EME1 incises on the 3′ side of ICL damage as previously hypothesized, we labeled the leading strand at the 3′ end of the ICL-damaged splayed arm by α-^32^P incorporation (Supplementary Figure S3). By annealing with different oligos on leading and/or lagging strands, 5′ ssDNA branch, 3′ ssDNA branch and static replication fork structures were created (Supplementary Figure S2). We next incubated the purified MUS81-EME1 with the DNA structures with ICL. If MUS81-EME1 incised the top leading strand at the junction site on the 3′ side of the ICL, a short DNA fragment would be expected. Surprisingly, no incision product below 74 nt could be detected with increasing amount of MUS81-EME1 (Figure 1). Instead, a much larger incision product band was observed with 3′ ssDNA branch and static replication fork structures (Figure 1, lanes 6–9). These data indicate that our MUS81-EME1 does incise the ICL-damaged DNA and has the same structure specificity for 3′ ssDNA branch and replication fork as previously reported (37–46), but it does not incise leading strand at the junction site on the 3′ side of the ICL damage as previously proposed.

### MUS81-EME1 incises the leading strand at the 5′ side of the psoralen ICL

To determine the exact incision site, we next labeled the 5′ ends of the leading and lagging strands separately (Supplementary Figure S3). Again, incubation of MUS81-EME1 with ICL substrates with 5′ end labeling on the lagging strand showed that the endonuclease did not react with splayed arm and 5′ ssDNA branch structures, but it effectively incised ICL-damaged 3′ ssDNA branch and replication fork (Supplementary Figure S4A, lanes 9–16). The incision was not on the splayed arm side of the lagging strand because only a large incision product was observed (Supplementary Figure S4A). As expected, MUS81-EME1 also did not incise undamaged DNA on the lagging strand (Supplementary Figure S4B).

**Figure 2.**
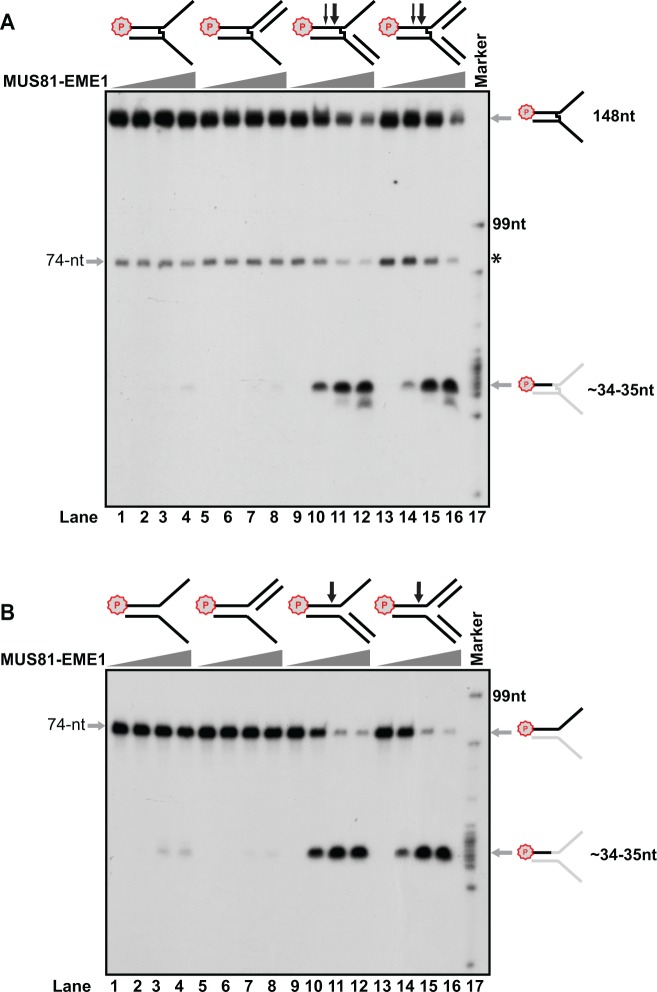
MUS81-EME1 incision on DNA substrates with 5′-end labeling on the leading strand. (**A**) Psoralen ICL-damaged substrates. (**B**) Undamaged structures. Titration of purified MUS81-EME1 (3 nM, 6 nM and 12 nM) on DNA substrates shown on the top of each gel. The schematic appearance of the products after incision was shown on the right. Letter P with a circle indicates γ-^32^P labeling by T4 polynucleotide kinase. Asterisk (74 nt) indicates a decayed and uncross-linked species. Arrows point to the incision sites and corresponding incision products.

Next, MUS81-EME1 was titrated with ICL substrates labeled at the 5′ end on the top leading strand. As shown in Figure 2, incision of the ICL-containing 3′ ssDNA branch and replication fork structures yielded a major band at ∼34–35 nt position and some smaller minor bands (Figure 2A, lanes 9–16). Because the distance between the 5′ end ^32^P labeling and the junction site was 39 nt, the predominant site of MUS81-EME1-mediated incision of ICL was calculated to be ∼4–5 nt away from the junction and located at the 5′ side of the ICL. MUS81-EME1 also incised undamaged controls at the similar site with slightly stronger activity (Figure 2, compare the remaining substrate bands located on the top of lanes 9–16 of B with those in lanes 9–16 of A). However, no smaller minor bands were observed with the undamaged DNA.

**Figure 3.**
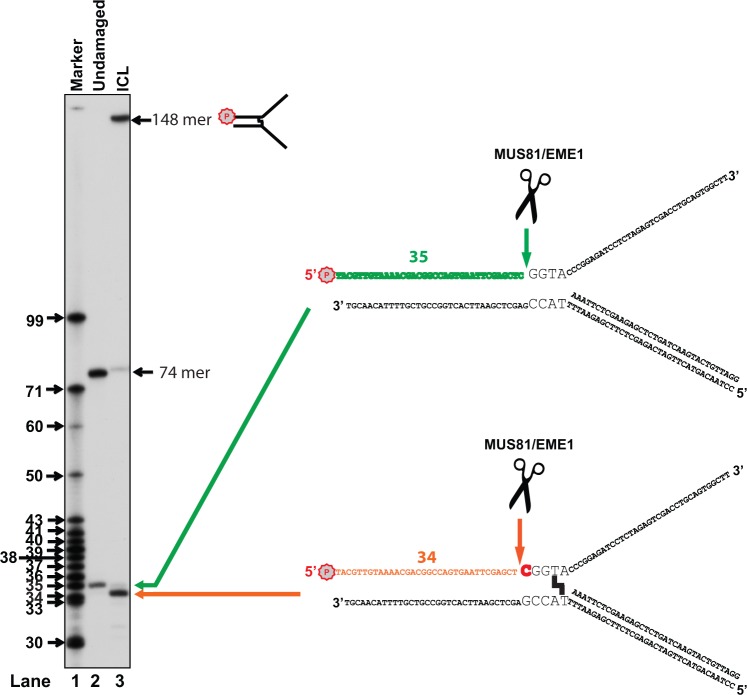
Psoralen ICL alters the MUS81-EME1 incision site. Lane 1: ^32^P labeled DNA markers shown in nucleotide on the left. A total 3 nM of purified MUS81-EME1 was incubated with the undamaged (Lane 2) and ICL-damaged 3′-ssDNA branch substrates (Lane 3). The sequence and schematic appearance of the products after incision were shown on the right. A 74-mer indicated a decayed and uncross-linked species. Letter P with a circle indicates 5′-^32^P labeling.

**Figure 4.**
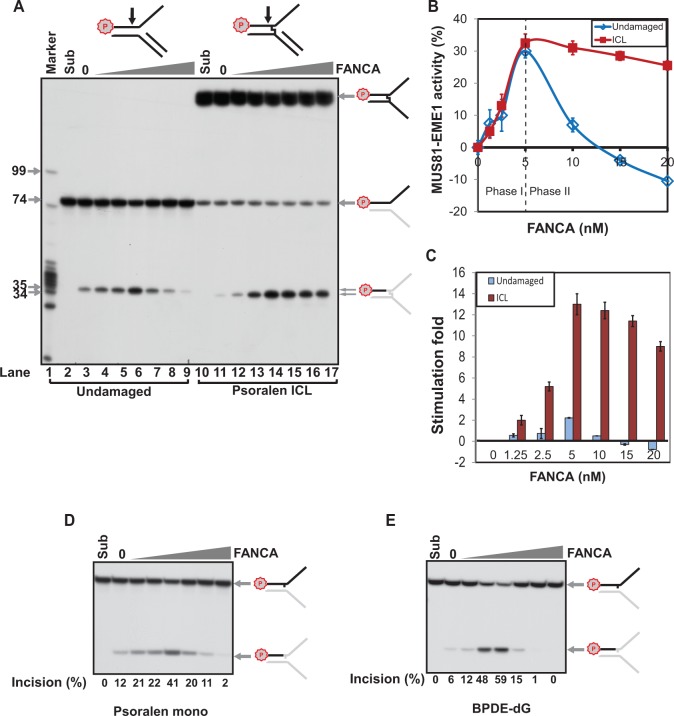
Effect of FANCA on MUS81-EME1-mediated DNA incision. (**A**) Undamaged and psoralen ICL-damaged substrates were incubated with 1.5 nM of MUS81-EME1, respectively, and an increasing concentration of FANCA (0, 1.25, 2.5, 5, 10, 15 and 20 nM). DNA markers are shown in nucleotide on the left. Same marker as described in Figure 3 was used. (**B**) Quantitation of three independent experiments. MUS81-EME1 activity was calculated as percentage of incision products out of the input substrates. The experiment without FANCA was normalized to 0 and used to calibrate all other experiments with the indicated amount of FANCA. Error bars: standard deviation. Dashed line: Phase I and Phase II border. (**C**) Fold changes of the MUS81-EME1 regulation by FANCA. The experiment without FANCA was arbitrarily normalized to 0 for fold change and used to calibrate all other experiments with the indicated amount of FANCA. Error bars: standard deviation. Psoralen mono-adducted (**D**) and a benzo(α)pyrene diolepoxide deoxyguanosine adducted 3′ ssDNA branch structures (**E**) were incubated with 1.5 nM of MUS81-EME1 and an increasing concentration of FANCA (0, 1.25, 2.5, 5, 10, 15 and 20 nM). The schematic appearance of the products after incision are shown on the right. Letter P with a circle indicates 5′-^32^P labeling.

During our test, we noticed a slight size difference between the major incision product from ICL substrates and the one from undamaged controls (Figure 4A). To define the difference, we analyzed the incision products by extensively running the reaction mixtures from ICL-damaged and ICL-undamaged DNA next to each other with a size marker. Figure 3 clearly showed that the major incision product from the ICL-damaged DNA is 34 nt in size, 1 nt shorter than the product from the undamaged control. This means MUS81-EME1 incises the psoralen ICL-damaged DNA 5 nt away from the fork junction, whereas it cuts the undamaged DNA 4 nt away (Figure 3).

These data establish that MUS81-EME1 unequivocally incises leading strand at the 5′ side of the psoralen ICL when located at the junction site and the psoralen damage affects the incision sites of MUS81-EME1.

### FANCA regulates MUS81-EME1-mediated DNA incision in a damage-dependent manner

Because FANCA interacts with DNA (28) and plays a role in ICL incision (29,30), we reasoned that FANCA may directly interact with ICL-unhooking DNA endonucleases such as MUS81-EME1 for more efficient ICL incision. To test this hypothesis, purified FANCA was titrated in the presence or absence of the psoralen ICL damage in the defined *in vitro* incision assay with suboptimal amount of MUS81-EME1 (1.5 nM) (Figure 4A and B). 

In the presence of ICL damage, FANCA dramatically stimulates ICL incision mediated by MUS81-EME1 up to 14-fold (Figure 4A and B, ICL and Figure 4C). Intriguingly, FANCA exerted a two-phase regulation of MUS81-EME1 activity in the absence of the ICL damage (Figure 4A, undamaged). In Phase I, increasing FANCA concentration up to 5 nM enhances MUS81-EME1 incision activity of undamaged DNA, although less efficiently than its effect on ICL-damaged DNA (Figure 4C, up to 3-fold). In Phase II, increasing FANCA concentration from 10 to 20 nM inhibits MUS81-EME1 activity on the undamaged DNA. MUS81-EME1 incision activity was abrogated at 20 nM FANCA (Figure 4A and B). Using 20 nM of FANCA and 1.5 nM MUS81-EME1, we also performed a time course experiment to further examine the extent to which FANCA stimulates or inhibits MUS81-EME1-mediated incision in the presence or absence of ICL (Supplementary Figure S5). The results clearly confirmed that FANCA stimulates MUS81-EME1 incision on the ICL-damaged DNA, and under the same condition it inhibits MUS81-EME1 activity in the absence of ICL. These observations demonstrate that FANCA functionally interacts with MUS81-EME1 to distinguish an ICL from undamaged DNA for damage-specific incision. 

It has been previously reported that MUS81 is involved in ICL incision but spares DNA damage that affects only one DNA strand (48). Inhibition of MUS81-EME1 activity by FANCA in the absence of DNA damage (Figure 4A and B) inspired us to hypothesize that FANCA might also be involved in the regulation of MUS81-EME1 activity on non-ICL DNA damage. To test this hypothesis, we performed the same FANCA titration using the psoralen thymine mono-adduct designed for the creation of the ICL. The psoralen mono-adduct is identical in sequence and overall structure to the ICL, only it was not exposed to ultraviolet A irradiation and consequently not allowed to form an ICL. Surprisingly, like on the undamaged DNA, FANCA stimulated or inhibited MUS81-EME1-mediated incision of the psoralen mono-adduct in a two-phase concentration-dependent manner (Figure 4D, compare the last lane with 20 nM of FANCA to the second lane without FANCA). Furthermore, experiments using a benzo(α)pyrene diolepoxide adducted deoxyguanosine, a damage that affects only one DNA strand (49), also showed that FANCA can stimulate or suppress MUS81-EME1 activity in a two-phase concentration-dependent manner (Figure 4E, compare the last two lanes with 15 and 20 nM of FANCA, respectively, to the second lane without FANCA). 

Overall, we demonstrated that FANCA directly participates in ICL-specific incision via the stimulation of MUS81-EME1 activity. Up- and downregulation of MUS81-EME1 activity by FANCA provides an important mechanism to uncover why it is possible for MUS81 to be involved in the incision of ICLs but not DNA damage that affects one DNA strand (48). 

### Both N- and C-terminals of FANCA are required for the regulation of MUS81-EME1

Because FANCA is a DNA binding protein, we next asked whether the damage-dependent regulation of MUS81-EME1 by FANCA is caused by its affinity to DNA. To test, we created two truncation mutants of FANCA, Q772X and C772-1455. Q772X is a Fanconi anemia disease-causing C-terminal truncation mutant. C772-1455 is the complementing C-terminal fragment of Q772X (Supplementary Figure S1). The DNA binding domain of FANCA is located at the C-terminal C772-1455 fragment (28). Therefore, it is conceivable to hypothesize that the C-terminal of FANCA confers the MUS81-EME1 regulating activity. 

**Figure 5.**
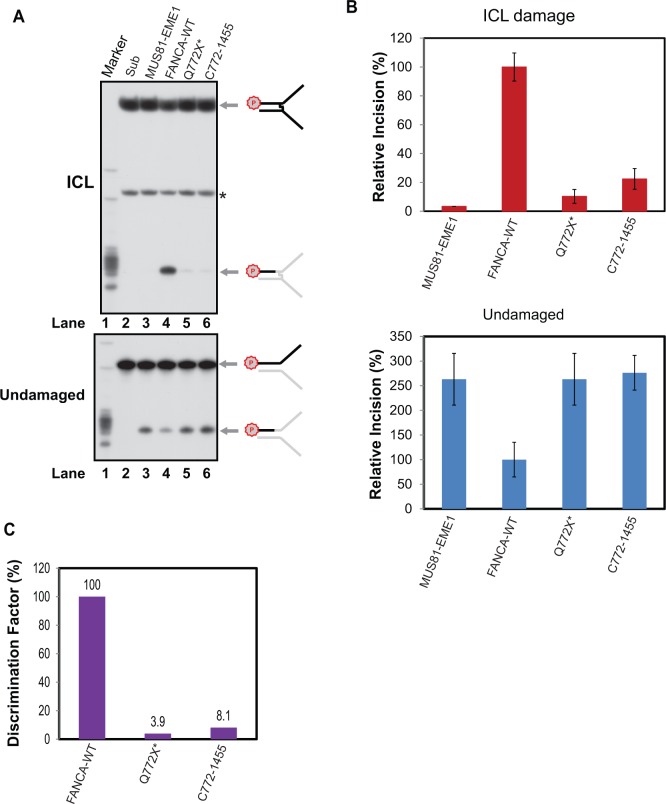
Effect of FANCA mutants on MUS81-EME1-mediated DNA incision. (**A**) Psoralen ICL-damaged (top) and ICL-undamaged DNA substrates (bottom panel) were incubated with 1.5 nM of MUS81-EME1 and 20 nM of purified WT and mutant FANCA proteins as indicated. DNA markers are shown on the left. The schematic appearance of the products after incision are shown on the right. Letter P with a circle indicates 5′-^32^P labeling. Asterisk indicates a decayed and uncross-linked species. (**B**) Quantitation of three independent experiments. Incision efficiency was normalized to WT-FANCA (arbitrarily assigned as 100%).**** Error bars: standard deviation. (**C**) Ability to discriminate ICL from undamaged DNA by FANCA mutants. The discrimination factor was calculated by dividing the relative incision rate of ICL of a protein in (B) by the relative incision rate of undamaged DNA of the same protein. The discrimination factor for FANCA was arbitrarily assigned as 100%.

Using 20 nM of protein, where WT-FANCA stimulates MUS81-EME1-mediated incision of ICL but inhibits incision of undamaged DNA (Figure 4A and B), we found that both mutants showed drastic reduction in stimulating MUS81-EME1 in the presence of ICL comparing with the WT protein (Figure 5A and B, compare lanes 5–6 with lane 4 in the ICL panel). Additionally, different from WT-FANCA, both the N- and C-terminals of FANCA did not inhibit MUS81-EME1 activity on undamaged DNA (Figure 5A and B, compare lanes 5–6 with lane 4 in the undamaged panel).

To further evaluate the functional application of the FANCA mutants, we created an incision discrimination factor for measuring the ability of FANCA in regulating MUS81-EME1-mediated incision of ICL damage versus undamaged DNA. Figure 5C clearly shows that both mutants in conjunction with MUS81-EME1 lost their ability to discriminate an ICL from undamaged DNA during incision. In summary, these data suggest that both N- and C-terminals of FANCA are critical for the regulation. 

### FANCA interacts with and recruits MUS81-EME1 to the ICL damage site

**Figure 6.**
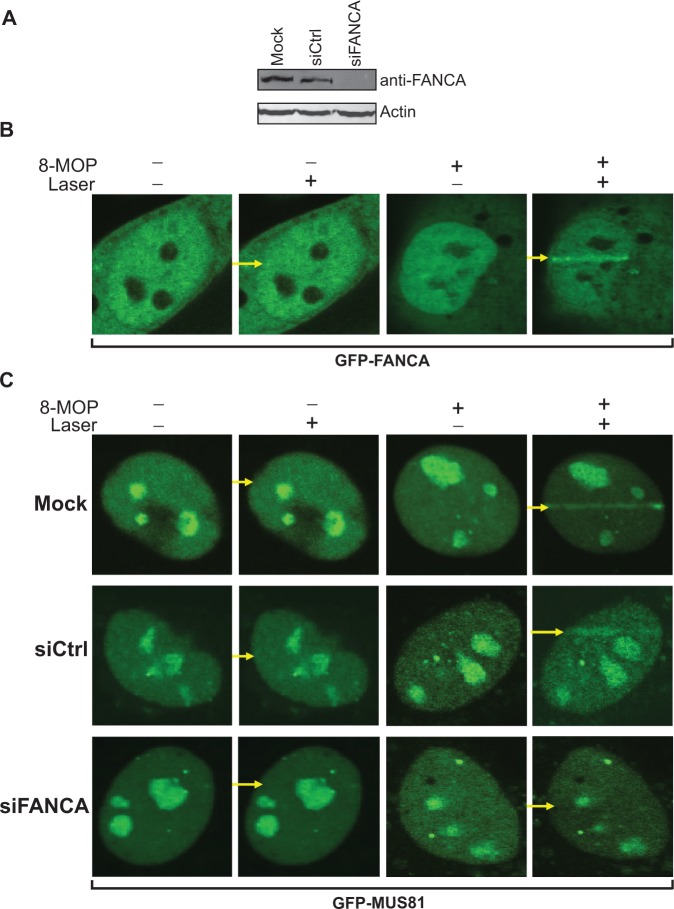
Interaction of FANCA and MUS81 on ICL damage in living cells. **** (**A**) Western blot of FANCA knockdown. U2OS cells were transfected with an ON-TARGET Plus SMART Pool siFANCA, a control siRNA (siCtrl), and dH_2_O (Mock) through lipofectamine. Forty-eight hours later, 50 µg of whole cell protein extract was prepared for western blot analysis using a FANCA-specific antibody. Actin is the loading control. (**B**) U2OS cells were transfected with GFP-FANCA and treated with 8-MOP and/or a 405 nm laser beam as indicated. Laser passing path is indicated by yellow arrows. The panel is representative of 25 examined nuclei of each treatment. 25/25 showed the laser-induced FANCA stripes in the presence of 8-MOP. (**C**) Mock, control siRNA and siFANCA treated U2OS cells were transfected with GFP-MUS81 and treated with 8-MOP and/or a 405 nm laser beam as indicated. Laser passing path is indicated by yellow arrows. The panel is representative of 25 examined nuclei of each treatment. 25/25 showed the laser-induced MUS81 stripes in the presence of 8-MOP for the mock and control treatment. 25/25 of siFANCA nuclei did not show the laser-induced MUS81 stripes in the presence of 8-MOP.

To examine whether FANCA interacts with MUS81-EME1 *in vivo*, we created site-specific ICL damages in living human cells. It has been reported that cells treated with 8-MOP form ICL damages after light activation (50,51). To determine the dynamics of FANCA responding to ICL damage, we expressed Green fluorescent protein (GFP)-FANCA in U2OS cells and visualized them through confocal microscopy. As expected, GFP-FANCA was expressed in both nucleus and cytoplasm (Figure 6B). The cells were then treated with either 8-MOP or low-energy 405 nm laser beam or a combination of both. FANCA did not respond to laser treatment indicating minimum damage formation by the laser alone (Figure 6B). Next, we pretreated the cells with 8-MOP and induced ICL formation through the laser beam irradiation. Surprisingly, only 2 min after laser treatment, FANCA was efficiently recruited to the lesion created in the path of the laser where ICLs formed (Figure 6B).

To investigate whether FANCA interacts with MUS81 on ICL damages, we then knocked down endogenous FANCA level by siRNA (Figure 6A) and transfected the knockdown U2OS cells with GFP-MUS81 before treatment with 8-MOP and/or laser (Figure 6C). As expected, GFP-MUS81 was only found in nucleus. Like FANCA, GFP-MUS81 did not respond to laser treatment, further proving that the laser produces minimal damage (Fig. 6C, 405 nm laser panel). After inducing ICL formation by both 8-MOP and laser, we observed effective MUS81 recruitment to the ICL damage sites when the cells were mock treated or treated with control siRNA. However, recruitment of MUS81 to the ICL site diminished when FANCA was successfully knocked down (Figure 6C, siFANCA panel). This result suggests that recruitment of MUS81 to ICL sites is dependent on FANCA and that FANCA functions upstream of MUS81 in the ICL repair pathway. 

## DISCUSSION

Heterodimeric DNA endonuclease MUS81-EME1 is known to be involved in incision of the ICLs in mammalian cells (48) and has been proposed to cut the leading strand at a replication fork junction site at the 3′ side of ICL damage (33–36). Using a defined psoralen ICL substrate, we have provided the first biochemical evidence that human MUS81-EME1 does incise ICL-damaged fork structures. However, unlike the previously proposed 3′ side cleavage models, MUS81-EME1 incises the leading strand at the 5′ side of an ICL lesion. This discrepancy was startling at first look. Nevertheless, further in-depth analysis revealed that our results are compatible with the previously published incision behavior and substrate specificity of MUS81-EME1. Similar to previous observations using yeast proteins, our results showed that purified human MUS81-EME1 incises 3′ ssDNA branch and replication fork structures 4 nt away from the junction site (37,52). This special activity is likely to lend MUS81-EME1 an ability to incise at the 5′ side of DNA across the ICL damage. Theoretically, cleavage to the 3′ side could happen only if the incision occurs long before the nascent strand reaches the ICL damage because of the incision activity of MUS81-EME1 on undamaged DNA.

Experimental evidence using *Xenopus* egg extracts and ICL-damaged plasmids showed that incision of ICLs happens after the replication forks in opposite directions converge at the ICL site (3,4). However, in some occasions, replication forks may not converge at the ICL site because of chromatin structures and/or relative distance between replication origins and the ICL damage. The excellent work done by using ICL-damaged plasmids may represent many but not necessarily all situations for replication fork stalling. In an occasion where the leading strand DNA synthesis pauses ∼24 nt upstream of the ICL, the DNA unwinding and lagging strand DNA synthesis may uncouple from the leading strand synthesis. The resulting DNA structure from this occasion resembles the ICL-damaged 3′ flap structure we used in our study. Nonetheless, studies from McHugh’s group provided strong evidence that DNA exonuclease SNM1A collaborates with endonuclease XPF-ERCC1 to initiate ICL repair (53). MUS81-EME1 may only act as an alternative mechanism when SNM1A and XPF-ERCC1 fail (34,53). 

**Figure 7.**
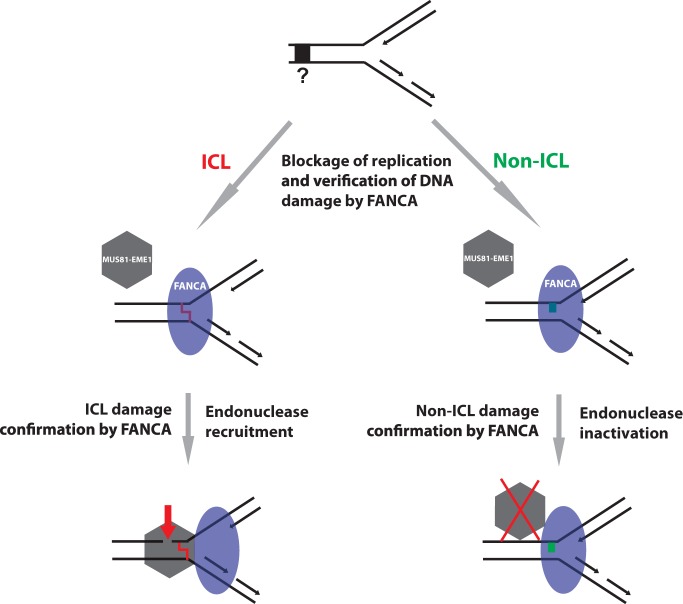
A working model for the regulation of MUS81-EME1 activity by FANCA. Blockage of DNA replication forks by an unknown damage (black box and question mark) initiates recruitment of FANCA (blue oval). FANCA recognizes ICL damage (red zigzag) and subsequently recruits MUS81-EME1 to incise the leading strand at the 5′ side of the ICL. If the damage only affects one DNA strand (green block) or there is no damage, FANCA will inactivate MUS81-EME1 to prevent unnecessary incisions. Red arrow: DNA incision; red cross: inactivation.

The most important discovery of our studies is that FANCA regulates MUS81-EME1-mediated DNA incision, positively and negatively, depending on the type of DNA damage. Enhancement of MUS81-EME1 activity in the presence of the psoralen ICL damage helps to repair the damage more effectively, and suppression of MUS81-EME1 in the absence of ICL damage helps in protecting replication forks from being attacked due to other DNA damage affecting only one strand. This in turn prevents the production of more deleterious damages such as DSBs. Both events are beneficial for the maintenance of replication forks. It is conceivable that the ICL damage is encountered and recognized by components of the replication machinery. The DNA binding activity of FANCA may serve to verify the presence of an ICL stalled replication fork. If an ICL is confirmed, FANCA will recruit and activate MUS81-EME1 for efficient and precise ICL incision (Figures. 6 and 7, ICL). If non-ICL damage is detected, FANCA will prevent MUS81-EME1 from incising on the stalled replication forks, a mechanism that is yet to be elucidated (Figures 4 and 7 non-ICL). This working model reconciles with our observations and explains why MUS81-EME1 promotes ICL unhooking yet avoids non-specific incision of undamaged or non–ICL-damaged forks (48). This scenario is similar to the damage recognition and verification steps in nucleotide excision repair where Xeroderma pigmentosum group C (XPC) recognizes DNA damage and Xeroderma pigmentosum group A (XPA)-Replication Protein A (RPA) verifies before incision to prevent unnecessary incisions (54). This process definitely requires both the DNA binding activity at the C-terminal and an unidentified function at the N-terminal of FANCA (Figure 5). Whether FANCA recognizes ICL and interacts with factors other than MUS81-EME1 for efficient ICL incision needs to be addressed in future studies.

It remains a mystery why low concentration of FANCA stimulates MUS81-EME1 activity in the absence of DNA damage, although the stimulatory activity is lower than in the presence of ICL (Figure 4). Because MUS81-EME1 is also involved in resolution of Holliday junction, which is a later step in the Fanconi anemia pathway of ICL repair (36,55), we speculate that FANCA may additionally be involved in regulation of Holliday junction resolution catalyzed by MUS81-EME1. It would be interesting to address whether and how FANCA affects MUS81-EME1-mediated incision of Holliday junctions. 

FANCM has been considered an ICL damage recognition factor because it can stabilize and remodel stalled replication forks, thus it may provide temporal and spatial access for the damage to be repaired (56,57). FANCM also appears to be required for assembly of the FA core complex onto chromatin and subsequent monoubiquitination of the FANCI–FANCD2 complex (8,33,58–65). Controversially, some other reports demonstrated that FANCM is not required for the formation of the eight-subunit core complex and FANCM null cells are only partially defective in damage-induced FANCD2 monoubiquitination (58,66,67). Evidence from FANCM^−^^/^^−^ knockout mice further demonstrated that FANCM may have a stimulatory but not essential role in monoubiquitinating FANCD2 (68). Furthermore, a direct interacting partner for FANCM-FAAP24 in the FA core complex has not been identified thus far, although FANCM-FAAP24 was originally identified through protein association in a FANCA-specific immunoprecipitation assay (22,60,69). Additionally, FANCM^−^^/^^−^ cells are sensitive to camptothecin, a topoisomerase inhibitor. Susceptibility to camptothecin is a unique feature identified only for downstream repair factors such as FANCD1/BRCA2 and FANCN/PALB2, but not for components of the FA core complex (66). In summary, these observations suggest that FANCM may act downstream of FANCD2, and therefore the upstream FA core complex may be recruited to DNA through other mechanisms. One of such mechanisms is likely through the DNA binding activity of FANCA (28). 

FANCA also interacts and colocalizes with XPF-ERCC1, another important ICL unhooking DNA endonuclease (70–73). Both MUS81-EME1 and XPF-ERCC1 interact with FANCP/SLX4, a newly identified Fanconi anemia protein. The FANCP-SLX1–XPF-ERCC1–MUS81-EME1 tri-endonuclease complex has been demonstrated to orchestrate nuclease actions during ICL incision and subsequent fork re-establishment through homologous recombination (6,34,36,74–79). It would be interesting to examine whether FANCA also regulates XPF-ERCC1 and the tri-endonuclease complex for efficient ICL incision as well as homologous recombination using our *in vitro* reconstitution system. 

In summary, this report provides novel insight into the incision behavior of MUS81-EME1 on ICL damage and establishes that FANCA contributes to the maintenance of replication forks by directly regulating the incision activity of MUS81-EME1 in a damage-dependent manner. 

## SUPPLEMENTARY DATA

Supplementary Data are available at NAR Online.

## FUNDING

National Institutes of Health (NIH) [HL105631 to Y.Z.], in part by [AG045545 to L.L.]; University of Pittsburgh Medical Center CMRF (to L.L.). Funding for open access charge: NIH.

*Conflict of interest statement*. None declared.

## Supplementary Material

Supplementary Data
