# Supplementary material for: Damage-dependent regulation of MUS81-EME1 by Fanconi anemia complementation group A protein
Source: Nucleic Acids Res. 2013 Oct 28;42(3):1671–83. doi: 10.1093/nar/gkt975 (PMC3919598; doi:10.1093/nar/gkt975)
Supplement: Supplementary Data [file supp_42_3_1671__index.html]

Damage-dependent regulation of MUS81-EME1 by Fanconi anemia complementation group A protein — Damage-dependent regulation of MUS81-EME1 by Fanconi anemia complementation group A protein — Supplementary Data 

# Damage-dependent regulation of MUS81-EME1 by Fanconi anemia complementation group A protein

## Supplementary Data

files

**Files in this Data Supplement:**

- Supplementary Data - pdf file
