# Supplementary material for: Damage-dependent regulation of MUS81-EME1 by Fanconi anemia complementation group A protein
Source: Nucleic Acids Res. 2013 Oct 28;42(3):1671–83. doi: 10.1093/nar/gkt975 (PMC3919598; doi:10.1093/nar/gkt975)
Supplement: Supplementary Data [file supp_gkt975_nar-02034-h-2013-File009.pdf]

## SUPPLEMENTARY INFORMATION

**Figure S1.** Purified proteins used in the study. The purified proteins were stained with Coomassie Brilliant Blue R-250 after SDS-PAGE. Asterisk indicates a hexahistidine tag at the N-terminus of the protein. Protein markers in kilo Daltons are indicated.

**Figure S2.** Creation of ICLs and different fork structures. T highlighted in blue font contains the synthetic psoralen. ICL is indicated by a red slash bar.

**Figure S3.** Labeling strategy of the ICL substrates. For labeling of the leading strand at the 3' end, a complementing oligo with a 5' protruding end was annealed as shown to allow incorporation of $\alpha$-$^{32}$P into the ICL substrate. For 5' end labeling, the indicated oligos were labeled by T4 polynucleotide kinase before ICL induction. T highlighted in blue font contains the synthetic psoralen. ICL is indicated by a slash bar. $^{32}$P is highlighted by red font.

**Figure S4.** MUS81-EME1 incision on DNA substrates with 5'-end labeling on the lagging strand. (**A**) Psoralen ICL damaged substrates. (**B**) Undamaged structures. Titration of purified MUS81-EME1 (3 nM, 6 nM, and 12 nM) on DNA substrates shown on the top of each gel. The schematic appearance of the products after incision are shown on the right. Letter P with a circle indicates $\gamma$-$^{32}$P labeling by T4 polynucleotide kinase. Asterisk (74-nt) indicates a decayed and uncrosslinked species. Arrows point to the incision sites and corresponding incision products.

**Figure S5.** Time course of MUS81-EME1 mediated incision in the presence or absence of FANCA. (**A**) Undamaged 3' flap structure was incubated with 1.5 nM of MUS81-EME1 in the presence of 20 nM FANCA (0, 1.25, 2.5, 5, 10, 15, and 20 nM) and with increasing incubation time (0, 1, 2, 5, 10, 20, 30 min). The schematic appearance of the products after incision were shown on the right. Letter P with a circle indicates $\gamma$-$^{32}$P labeling. An arrow points to the incision sites and corresponding incision products. (**B**) Psoralen ICL damaged 3' flap structure was

incubated with 1.5 nM of MUS81-EME1 in the presence of 20 nM FANCA and with increasing incubation time (0, 1, 2, 5, 10, 20, 30 min). The schematic appearance of the products after incision were shown on the right. Letter P with a circle indicates γ-$^{32}$P labeling. Asterisk indicates a decayed and uncrosslinked species. An arrow points to the incision sites and corresponding incision products. (**C**) Quantitation of three independent experiments for (A). MUS81-EME1 activity was calculated as % of incision products out of the input substrates. Error bars: standard deviation. (**D**) Quantitation of three independent experiments for (B). MUS81-EME1 activity was calculated as % of incision products out of the input substrates. Error bars: standard deviation.

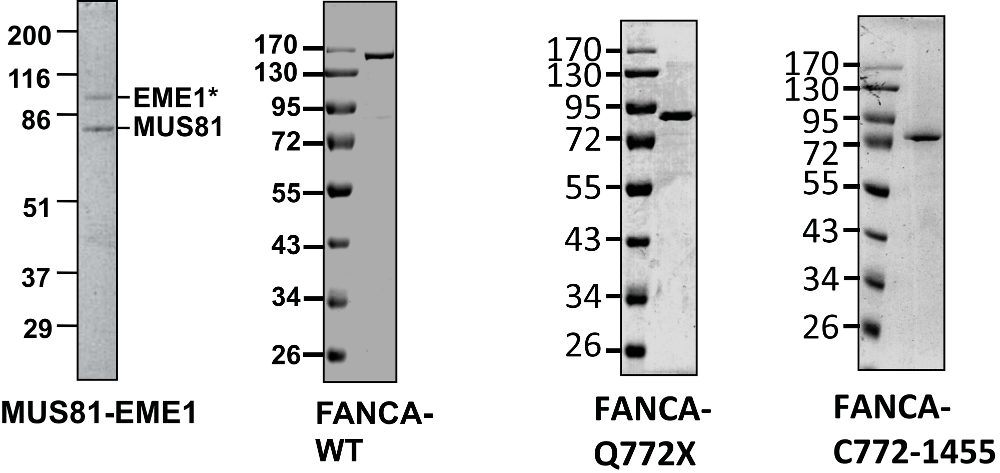

200
116
86
51
37
29
EME1*
MUS81
MUS81-EME1
170
130
95
72
55
43
34
26
FANCA-WT
170
130
95
72
55
43
34
26
FANCA-Q772X
170
130
95
72
55
43
34
26
FANCA-C772-1455


Figure S1

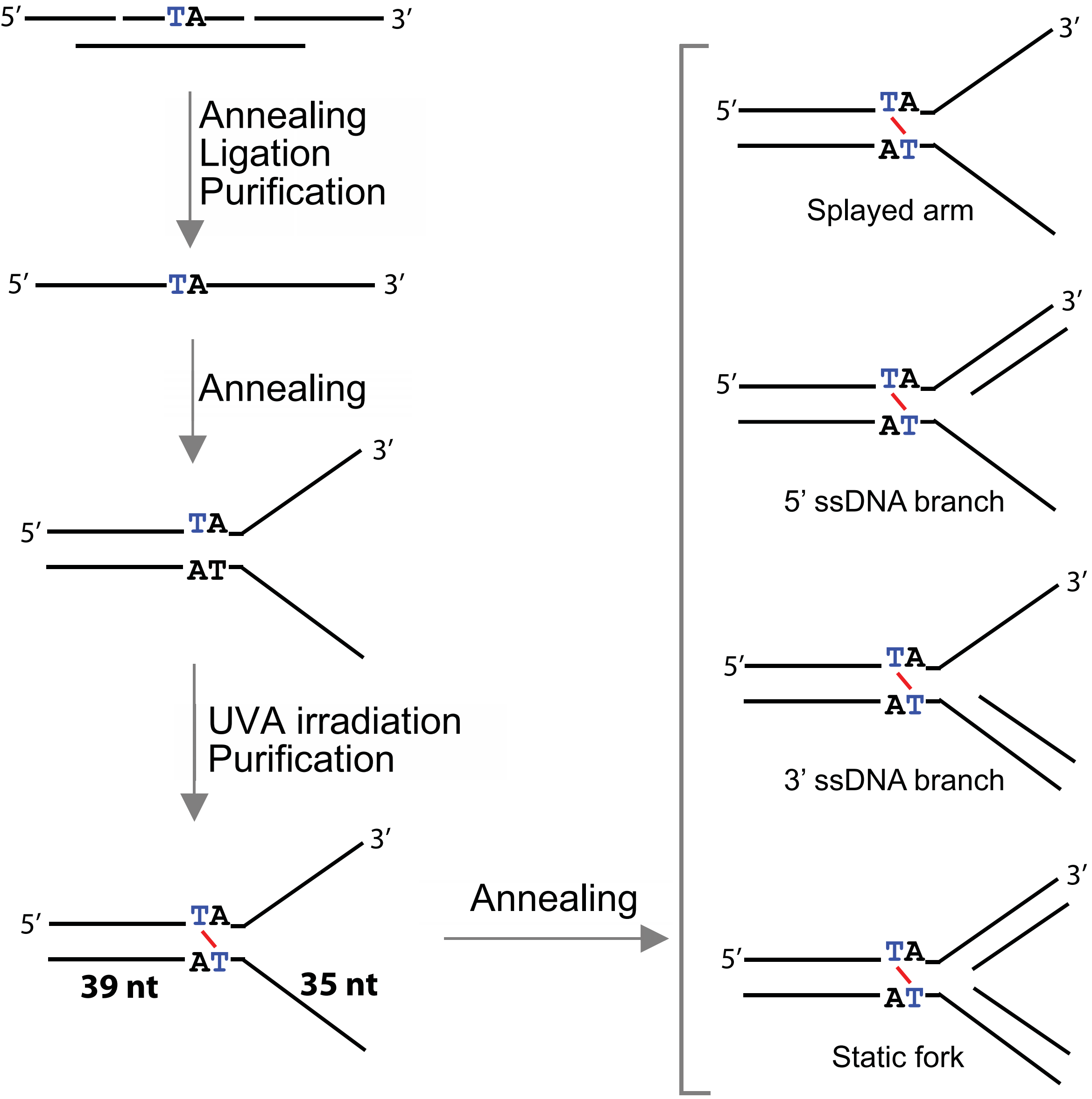
5′
TA
3′
Annealing
Ligation
Purification
5′
TA
3′
Annealing
3′
5′
TA
AT
UVA irradiation
Purification
3′
5′
TA
AT
39 nt
35 nt
Annealing
3′
5′
TA
AT
Splayed arm
3′
5′
TA
AT
5’ ssDNA branch
3′
5′
TA
AT
3’ ssDNA branch
3′
5′
TA
AT
Static fork

Figure S2

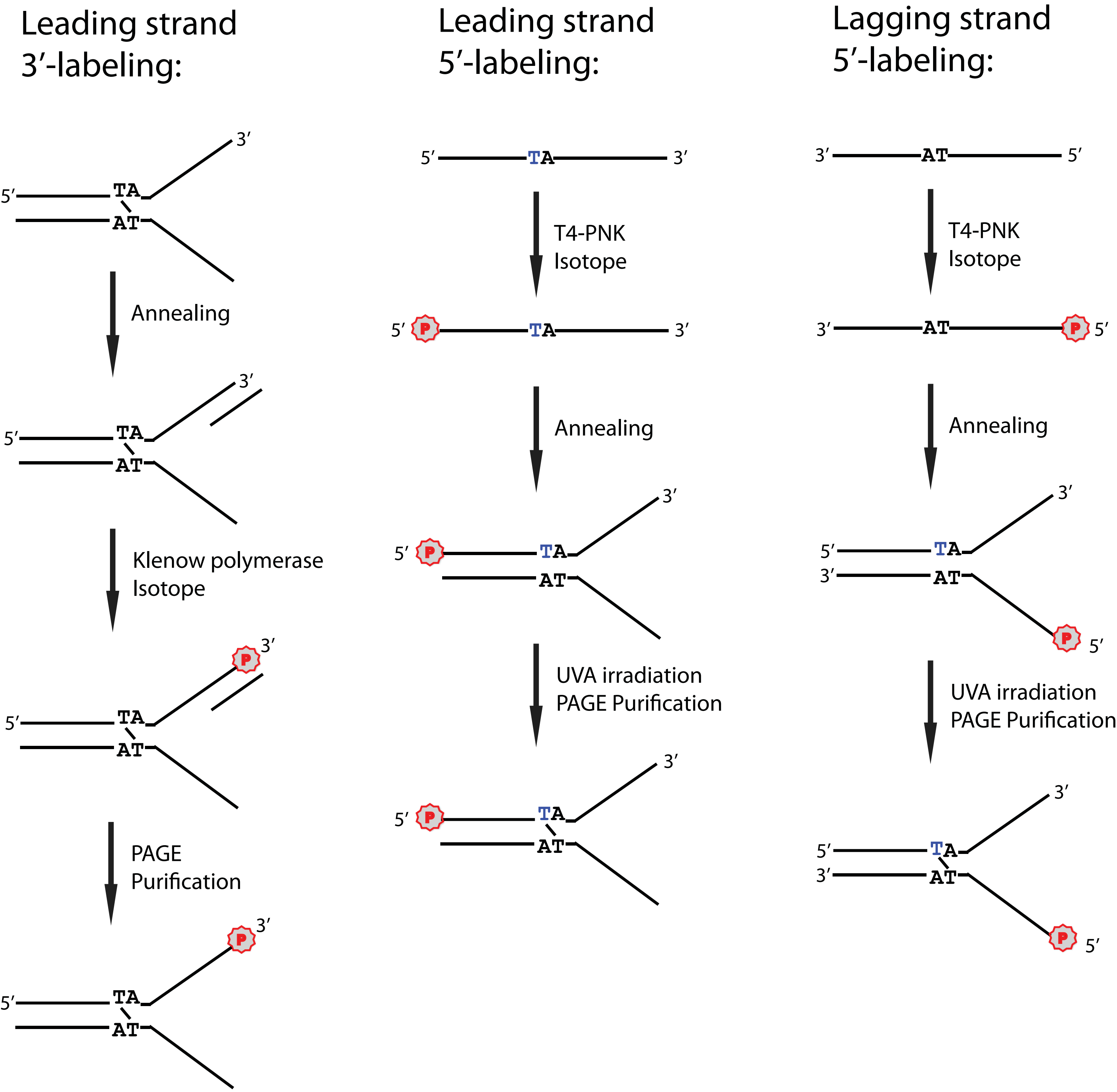
Leading strand 3′-labeling:
3′
5′
TA
AT
Annealing
3′
5′
TA
AT
Klenow polymerase
Isotope
P
3′
5′
TA
AT
PAGE
Purification
P
3′
5′
TA
AT
Leading strand 5′-labeling:
5′
TA
3′
T4-PNK
Isotope
5′
P
TA
3′
Annealing
3′
5′
P
TA
AT
UVA irradiation
PAGE Purification
3′
5′
P
TA
AT
Lagging strand 5′-labeling:
3′
AT
5′
T4-PNK
Isotope
3′
AT
P
5′
Annealing
3′
5′
TA
3′
AT
P
5′
UVA irradiation
PAGE Purification
3′
5′
TA
3′
AT
P
5′

Figure S3

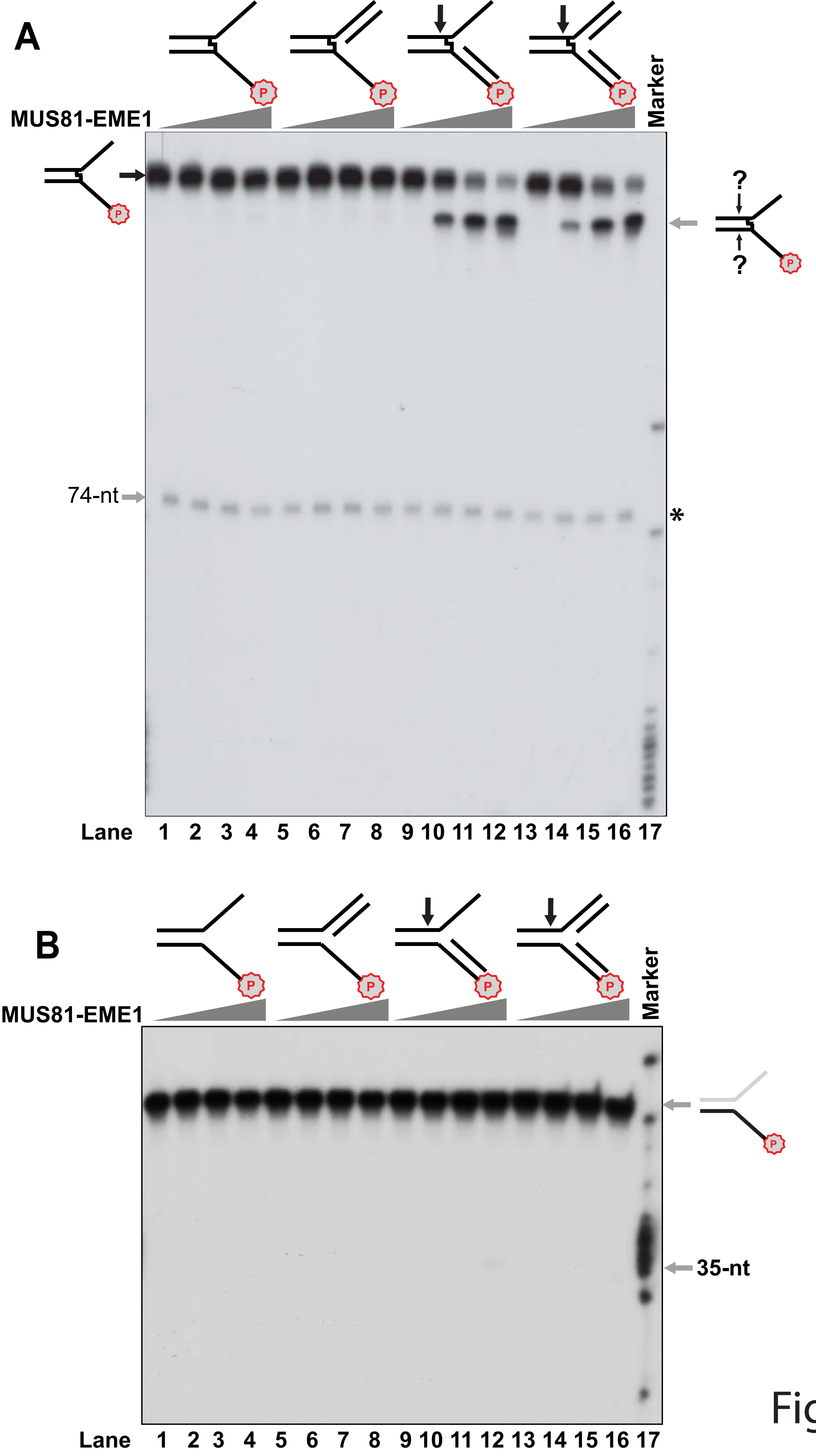
A
MUS81-EME1
Marker
74-nt
*
?
?
P
Lane 1 2 3 4 5 6 7 8 9 10 11 12 13 14 15 16 17
B
MUS81-EME1
Marker
35-nt
P
Lane 1 2 3 4 5 6 7 8 9 10 11 12 13 14 15 16 17

Figure S4

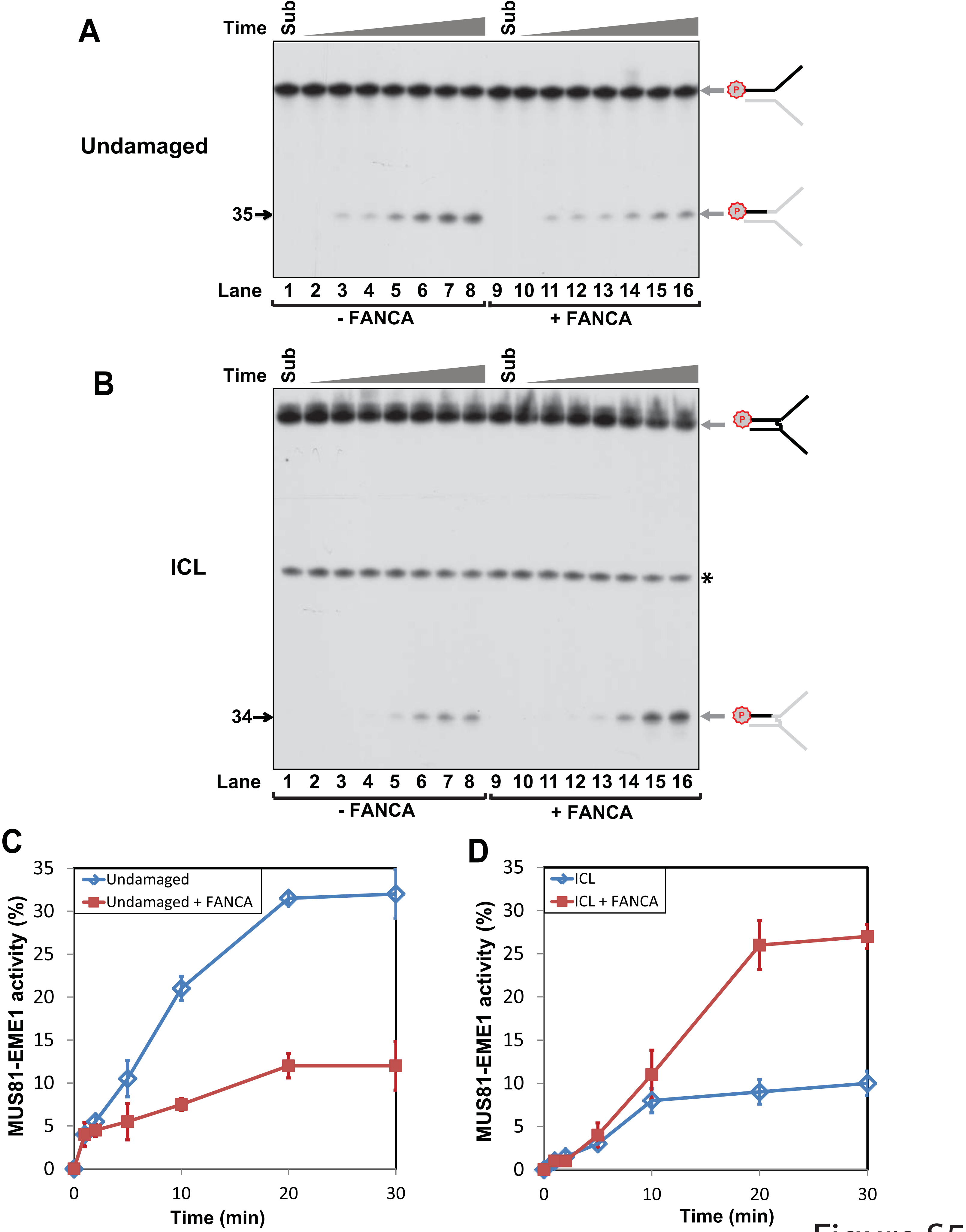

A
Time
Sub
Sub
Undamaged
35
Lane
1 2 3 4 5 6 7 8 9 10 11 12 13 14 15 16
- FANCA
+ FANCA
B
ICL
34
*
C
MUS81-EME1 activity (%)
Undamaged
Undamaged + FANCA
Time (min)
D
ICL
ICL + FANCA


Figure S5
